# Evaluation of Feline Permanent Canine Tooth Mineral Density Using Micro-Computed Tomography

**DOI:** 10.3390/vetsci10030217

**Published:** 2023-03-12

**Authors:** Graça Silva, Pedro S. Babo, Jorge Azevedo, Manuela E. Gomes, Carlos Viegas, João F. Requicha

**Affiliations:** 1 Department of Veterinary Sciences, University of Trás-os-Montes e Alto Douro, Quinta de Prados, 5000-801 Vila Real, Portugal; 2 3B’s Research Group, I3Bs—Research Institute on Biomaterials, Biodegradables and Biomimetics, University of Minho, 4805-017 Guimarães, Portugal; 3 Department of Animal Science, University of Trás-os-Montes e Alto Douro, Quinta de Prados, 5000-801 Vila Real, Portugal; 4 Veterinary and Animal Research Centre (CECAV), University of Trás-os-Montes e Alto Douro, Quinta de Prados, 5000-801 Vila Real, Portugal; 5 Associate Laboratory for Animal and Veterinary Sciences (AL4AnimalS), Portugal

**Keywords:** cat, mineral density, canine tooth, micro-computed tomography

## Abstract

**Simple Summary:**

The tooth is composed of three mineralized tissues: enamel, dentin, and cementum. Micro-computed tomography (mCT) is an imaging technology based on X-rays that allows non-invasive morphological and quantitative analysis. The present work aimed to determine the relative mineral density (MD) of the feline tooth using mCT. The studied sample consisted of nine canines extracted from European Shorthair cats due to medical indication. These teeth were evaluated through dental radiography and, using mCT, the values of MD of the root of each tooth and of specific segments corresponding to the coronal, middle, and apical thirds of the root were determined. Mean MD of root tissues was 1.374 ± 0040 g·cm^−3^, and of hard root tissues 1.402 ± 0.035 g·cm^−3^. The study of MD could become an ancillary method for the diagnosis and characterization of dental pathology.

**Abstract:**

The tooth is made up of three mineralized tissues, enamel, dentin, and cementum, which surround a non-mineralized tissue called the dental pulp. Micro-computed tomography (mCT) is an imaging technology based on X-rays that allows non-invasive visualization of objects at a microscopic scale, according to their radiopacity and in three dimensions (3D). Likewise, it allows the subsequent execution of morphological and quantitative analysis of the objects, such as, for example, the determination of the relative mineral density (MD). The present work aimed to describe the MD of feline teeth using mCT. The studied sample consisted of four European Shorthair cats, from which nine canine teeth were extracted per medical indication. These teeth were evaluated through dental radiography before and after their extraction. Using mCT and the CTAn software, the values of the relative mineral density of the root of each tooth and of specific segments corresponding to the coronal, middle, and apical thirds of the root were determined. Mean MD of root tissues was 1.374 ± 0040 g·cm^−3^, and of hard root, tissues was 1.402 ± 0.035 g·cm^−3^. Through mCT, it was possible to determine the mean MD values of feline canine teeth. The study of MD could become an ancillary method for the diagnosis and characterization of dental pathology.

## 1. Introduction

The healthy feline tooth is composed of the crown, the visible supragingival portion, and the root, the hidden subgingival portion, lodged in the alveolus. A central cavity is located within the tooth—called the pulp canal (in the root segment) and pulp chamber (in the crown segment). A soft tissue named dental pulp, consisting of nerves, blood vessels, and connective tissues, which provides nourishment and sensation to the tooth, is contained within the pulp canal and chamber. The pulp is surrounded and therefore protected by two hard mineralized layers: an inner layer composed of dentin, which is found both in the crown and root, and a thin outer layer composed of enamel in the crown and cementum at the root, which is separated at the level of the dental neck by the cementoenamel junction [1].

Hard mineralized dental tissues have a large amount of inorganic material. These values are 90% in the case of enamel, the hardest tissue in the body, 70% in dentin, the main tissue of adult teeth, and 65% in cementum, a mineralized connective tissue similar to bone. Calcium hydroxyapatite crystals are the main inorganic substance in the mineralized tissues, and small amounts of calcium carbonate, sodium, potassium and magnesium, carbon dioxide, and varying amounts of phosphorus and fluorine are also found. The remaining components of dentin and cementum are water, 10%, and 12% respectively, and organic substances, 20% and 23% respectively. In enamel, the remaining 10% includes water and organic substances [2].

One of the most recent imaging technologies with interest in clinical diagnosis and research in Dentistry is X-ray micro-computed tomography (mCT). This is a non-invasive method that allows the acquisition of high-resolution 3-dimensional (3D) images, at a microscopic scale, of both structure and density (or concentration), without destroying the tissue to be examined. Because cutting the biopsied sample is not required, the use of micro-CT will not impede subsequent histological analysis [3,4].

The mode of action of mCT is similar to that of CT, with the main differences being the focal point of the X-ray, which is smaller and therefore provides higher resolution images, and the fact that the source of the X-ray and the detector remain in the same position during the acquisition, with the object rotating around an axis. Two-dimensional X-ray projections are made for each angular step and, as with CT, a three-dimensional reconstruction of the examined object is performed, based on the acquired two-dimensional projections [5].

The use of mCT in human dentistry has increased with the evolution of technology, namely in scientific studies, to analyze the mineral density (MD) of enamel and dentin, to evaluate cavitary lesions involving these tissues, as well as to study microleakage in fillings and fissure sealants, to measure cortical bone density, to assess root canal morphology, and to compare the accuracy of root canal instrumentation and obturation. The ability to generate images with the micrometric resolution is particularly useful in dentistry, given the size and mechanical characteristics of the tissues that restrict the use of other diagnostic techniques with comparable sensitivity in vivo. Thus, mCT makes it possible to accurately determine characteristics such as enamel thickness, dentin, and pulp chamber volume and to assess the structure and volume of the root canal system at different levels [5].

In Veterinary Medicine, mCT has become an ex vivo tool for biopsies, valuable for research as well as for clinical diagnosis [5]. It has also proven to be effective as an in vivo diagnostic technique for visualizing small anatomical changes (30 mm) in rodents and lagomorphs [4]. However, the X-radiation used for mCT acquisitions and the acquisition time, in the order of tens of minutes, has a high mutagenic potential. The sample size is also a limiting factor. Typically, a benchtop mCT instrument has a sample chamber with a maximum dimension of 3 × 3 × 3 cm [4].

Recently, mCT equipment has been developed that allows overcoming the limitations of conventional benchtop equipment, namely reducing the acquisition time to just a few seconds, thus reducing the radiation to which the sample is exposed, allowing for simpler anesthesia protocols for in vivo acquisitions, with larger sample chambers, allowing the evaluation of small live animals and maintaining a high resolution (less than 2 µm/pixel) [6].

Mineral density is a measure of the amount of minerals present in any given volume of sample. Thus, the study of MD provides information about the mechanical properties of tissues. As previously mentioned, calcium hydroxyapatite, the main constituent mineral of the hard tissues of the tooth, is responsible for its stiffness [7]. mCT and light-induced fluorescence quantitative digital imaging are two recent methods suitable for determining the mineral content of human teeth [8].

In healthy human teeth, by using mCT, it was determined that the mean MD of the enamel varies between 2.84 and 3.31 g·cm^−3^ and has a tendency to gradually decrease towards the CEJ, while the mean MD of the dentin varies between 1.72 and 1.95 g·cm^−3^ [7]. In human teeth with enamel hypomineralization, also using mCT, it was found that the average MD of this tissue varies between 1.91 and 2.29 g·cm^−3^ [9]. Shahmoradi and colleagues found that, in teeth with cavitary lesions, the average MD of the enamel varies between 1.41 and 2.67 g·cm^−3^ [10].

In Veterinary Medicine, to our knowledge, there are no studies on MD of dental tissues in canine and feline species. However, a study was recently published that integrated the measurement of MD of the alveolar bone in dogs using mCT [11]. The determination of feline teeth MD using the proposed technique could be helpful in the characterization of dental pathology in cats, such as developmental abnormalities such as enamel hypoplasia or acquired ones such as odontoclastic tooth resorption, or neoplastic lesions with dental involvement.

Tooth resorption is a common nosological entity in domestic cats and presents itself as a defect in hard tissues (enamel, dentin, and cementum), due to the progressive loss of calcified dental tissue by the action of odontoclasts [12]. The main physiological role of odontoclasts is to promote root resorption of deciduous teeth and the hard tissues surrounding their roots, allowing the occurrence of tooth eruption of permanent teeth. In adult cats, these cells, due to still unknown causes, become deregulated and destroy dental tissues [12]. Several etiological factors have been suggested as the cause of this disease, such as chronic oral inflammation (periodontal disease and feline chronic gingivostomatitis; infectious diseases, namely those caused by the feline immunodeficiency virus (FIV) and the feline leukemia virus (FeLV) [13]; hypervitaminosis D with changes in calcium metabolism [14], mechanical trauma [15], or the breed of the animal, although none of these factors has been confirmed as the direct cause of resorption [16].

There are two types of root resorption, internal and external. Internal root resorption starts at the pulpal surface, i.e., at the inner surface of the root, and extends towards the outer surface of the tooth. It occurs when the integrity of the odontoblast layer (the outermost layer of the pulp tissue) is affected, usually as a consequence of pulpitis [17,18]. External or inflammatory root resorption is more frequent than internal root resorption and usually begins on the outer surface of the root or in the cementoenamel junction, progressing toward the interior of the tooth. It can be a consequence of any injury to the periodontal ligament or cementoblast layer, which causes subsequent loss of the root structure of the tooth and often ankylosis between the tooth and the alveolar bone [12,17].

Thus, this study aimed to investigate this parameter using micro-computed tomography and evaluate the soundness of this technology as a diagnosis tool in Veterinary Dentistry.

## 2. Materials and Methods

### 2.1. Studied Samples

The studied samples consisted of nine canine teeth extracted from four cats (*Felis catus*) at the Service of Dentistry and Oral Surgery of the Veterinary Hospital of the University of Trás-os-Montes and Alto Douro (Vila Real, Portugal). The extracted teeth were used after informed consent was obtained from the animals’ tutors.

All cats were male European Shorthair with ages ranging from two to eight years. Eight teeth were extracted within the surgical treatment of feline chronic gingivostomatitis and one was due to mandible fracture. At the time of the clinical examination, radiographic images of both maxillary and mandibular canine teeth were obtained with a dental X-ray generator (Triumph, Germany) and an image plate scanner (CR 7 Vet, Durr Dental, Bietigheim-Bissingen, Germany), by using the bisecting technique with 68 kV, 4 mA and an exposure time of 0.20 ms.

**Figure 1:**
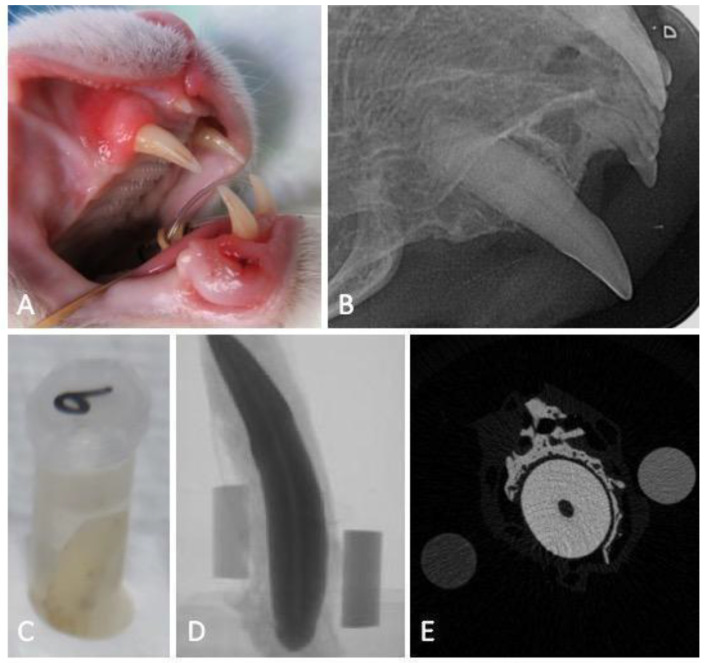
Flowchart of the experimental work: macroscopic image of a maxillary canine tooth obtained during the clinical examination before extraction (**A**), the respective intraoral dental radiograph revealing no signs of tooth resorption (**B**), sample storage (**C**) and micro-computed tomography projection (**D**) and transversal cut at the tooth root level (**E**), accompanied with the 0.25 and 0.75 g of HAp·cm^−3^ phantoms.

The studied teeth were considered healthy, based on the fact that no clinical (enamel defects, cavities, discolorations, fractures) or radiographic evidence of any feline dental disease were found. Upon extraction, teeth were immersed in a 10% neutral buffered formalin solution (Panreac AppliChem GmbH, Darmstadt, Germany) and stored at room temperature until the analysis (Figure 1A–C).

### 2.2. Micro-CT Image Acquisition Protocol

Feline canine teeth images were acquired in a humid environment, to avoid the formation of desiccation artifacts, as recommended by Bruker (Kontich, Belgium). For this, the teeth were wrapped in absorbent paper, soaked in mili-Q water (Ultrapure Lab Water, Merk Life Science S.L.U., Algés, Portugal) inserted, and adjuste d to the walls of a transparent polypropylene tube (15 × 58 mm) to avoid oscillations in the acquisition. Two phantoms of known MD, 0.25 and 0.75 g of HAp·cm^−3^, were wrapped in the absorbent paper together with the tooth for further the calibration and analysis of the tooth MD. The samples were digitized using a high-resolution X-ray mCT instrument (Skyscan 1272, Bruker, Belgium). A series of 2D projections with an isotropic resolution of 16.34438 µm per voxel was acquired by irradiation with penetrating X-rays, using a voltage of 70 kV, a current of 142 µA, and a 0.5 mm thick aluminum filter, in a 360° rotation amplitude with a rotation step of 0.45°, with an exposure time of 2660 ms per projection (Figure 1D,E).

### 2.3. Mineral Density Analysis Protocol and Reconstruction of 3D Images

**Figure 2:**
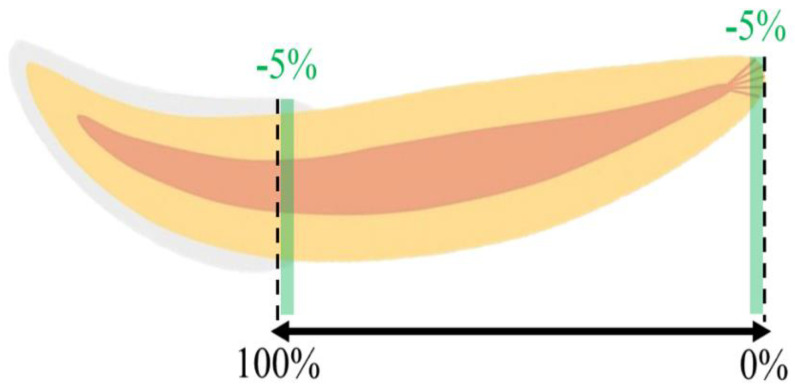
Schematic representation of the studied region of the tooth.

The images were reconstructed in NRecon software (version 1.6.6.0, Bruker, Belgium), correcting misalignment, ring artifacts, and beam hardening effects as needed. The analysis was then performed using the CTAn software (version 1.4, Skyscan). In each studied tooth, a region of interest (ROI) was manually defined as a circle centered on the root canal, to obtain a volume of interest that corresponded to the totality of the root, from the most apical end to the level of the CEJ where the enamel made up 50% of the circumference of the tooth neck. Considering the specific anatomy of the cat’s tooth, and to avoid the interference of irregularities in the apical and coronal portions in the analysis, the 5% apical and coronal terminals were excluded (Figure 2). In addition, three segments of the studied volume of interest were defined, relating to the coronal, middle, and apical thirds of the tooth, the first including a portion of the cementum-enamel junction. The obtained volumes of interest were the essential basis for the quantitative analyses, described below.

After calibrating the MD by integrating the average Hounsfield units (HU) of the volume of interest corresponding to each phantom, and their nominal MD, the MD of each sample was analyzed. In the first analysis, a radiopacity threshold was defined and included the entire volume of the dental tissues, allowing the global variation of the MD of the tooth (“total tissues”). In a second analysis, a radiopacity threshold was defined to allow the assessment of MD only in the volume of mineralized tissues (“hard tissues”). The MD was expressed in the density unit of g HAp·cm^−3^. The 3D models were generated in the CT VOX software (version 2.3.0 r810, SkyScan, Bruker, Belgium).

### 2.4. Statistical Analysis

This analysis was carried out in the “total tissues” and “hard tissues” of the studied region of the tooth. Due to the preliminary analysis of the data obtained, since the normality and homogeneity of variances had not been verified, Wilcoxon non-parametric test was performed to compare the MD between total tissues and hard tissues (JMP v.16.0, 2021, SAS Institute Inc., Cary, NC, USA). Those with *p* < 0.05 were considered statistically significant.

## 3. Result

### 3.1. Tridimensional Morphology

**Figure 3:**
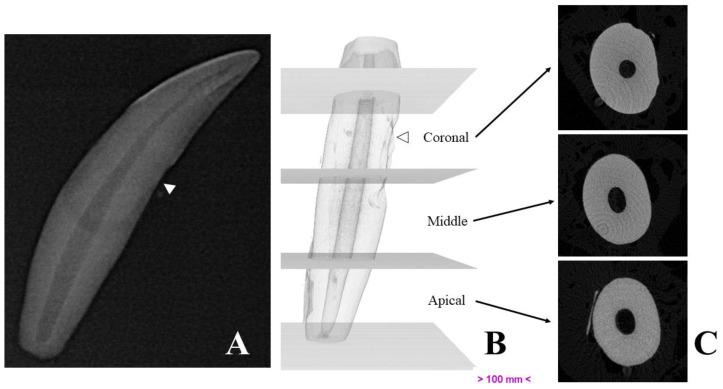
Representative images of a studied canine tooth. (**A**)—Radiography. (**B**)—Three-dimensional reconstruction, divided into thirds. (**C**)—Cross-sections obtained by micro-computed tomography. White arrows show drill marks made during tooth extraction.

**Figure 4:**
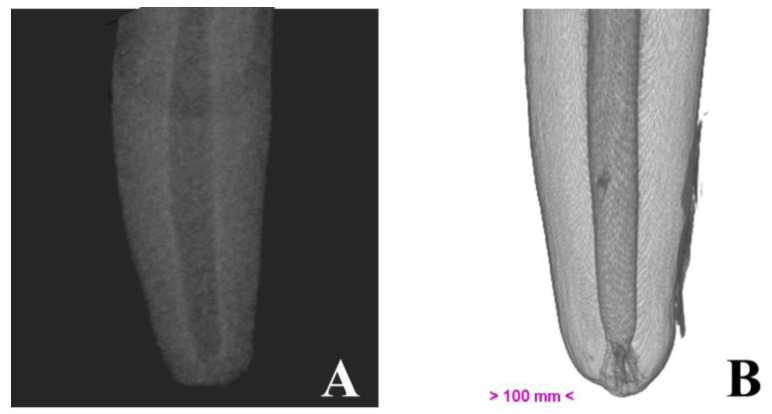
Illustrative image of the canine root, obtained by dental radiography (**A**) and by micro-computed tomography (**B**).

Micro-computed tomography allowed us to visualize dental structures which cannot be identified as effectively on dental radiography, with a rotation amplitude of 360° (Figure 3). The limits of the pulp canal can be observed in high detail. The apical delta at the bottom of the pulp canal, which is not visible on dental X-ray, is shown with great precision on mCT images (Figure 4).

### 3.2. Mineral Density

**Figure 5:**
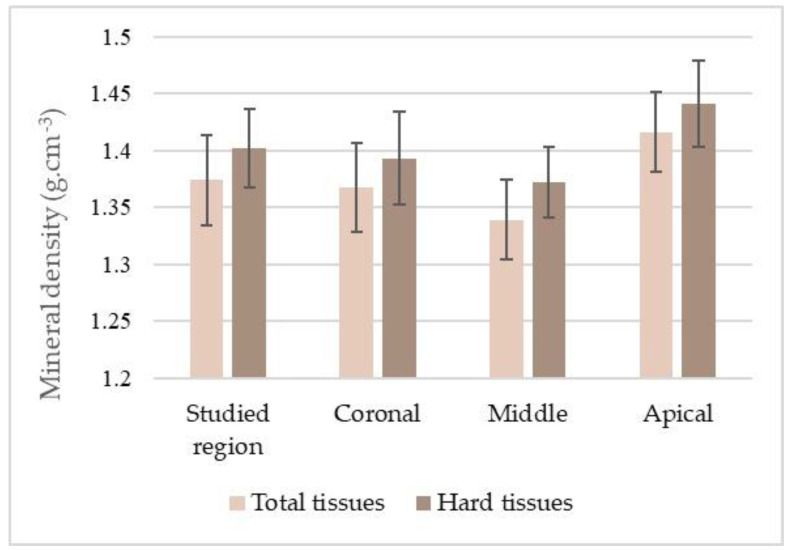
Distribution of the mineral density (mean and standard deviation) of the total tissues and hard tissues of the studied region of the tooth and its thirds.

Mean MD values of the total tissues were 1.374 ± 0040 g·cm^−3^, and of hard tissues was 1.402 ± 0.035 g·cm^−3^. Statistical analysis confirmed the significance of the differences between the MD values in the total tooth and in the coronal, middle, and apical thirds (Table 1; Figure 5).

## 4. Discussion

The present work was developed to study the MD of a cat’s teeth using the micro-computed tomography technique. Thus, it was intended to propose the determination of MD in the characterization of dental pathology in cats.

Mineral density analysis was performed using mCT, but its measurement would be also possible with the nondestructive quantitative light-induced fluorescence-digital (QLF-D) imaging method [8]. Although scanning electron microscopy (SEM), X-ray diffraction (XRD), energy-dispersive X-ray spectrometer (EDS), and inductively coupled plasma optical emission spectrometry (ICP) do not allow MD analysis, these methods are capable of quantifying the mineral content of the tooth [19,20,21].

The measurement of MD using mCT is a common practice in the field of orthopedics [22,23]. In a study conducted by Aerssens and colleagues, differences in bone mineral density were found in humans (0.178 g·cm^−3^), dogs (0.340 g·cm^−3^) and cows (0.449 g·cm^−3^) [24]. However, the use of this method in dental tissues is rare, and, to date, there are no studies related to this topic in companion animals, which makes the present study innovative and exploratory.

The obtained results allowed us to determine that the mean relative MD of the hard tissues of a healthy (with no clinical and radiographic signs of disease) cat tooth is 1.402 (±0.040) g·cm^−3^, with the maximum value obtained being 1.451 g·cm^−3^ and the minimum value 1.341 g·cm^−3^. Hayashi-Sakai et al. (2019) determined, using mCT, that the mean MD of dentin of a healthy human tooth varies between 1.72 and 1.95 g·cm^−3^ [7].

Upon extraction, the solution and storage time of the tooth pieces can affect properties of adsorption, diffusion, and dissolution, and therefore possibly alter the physical properties of dentin [25,26]. It is recommended to minimize these changes to preserve tissue properties, for standardization and reproducibility of results. Therefore, in the present work, teeth were stored in a 10% neutral buffered formalin solution as described by Jameson and collaborators [27]. In addition, Hayashi-Sakai and colleagues (2019) submerged the teeth in 0.1% chloramine-T, while Tomaszewska and collaborators (2018) used a 5.25% sodium hypochlorite solution to remove superficial organic residues [7,28].

In our study, it was not possible to characterize enamel MD, since the dimensions of the polypropylene tube inserted in the mCT equipment did not allow it to fit the entire tooth, thus excluding the dental crown for the analysis. In the future, more advanced mCT equipments capable of evaluating whole dental samples and with higher resolution will be useful to distinguish contiguous mineralized tissues (dentin, enamel, and cementum) which can present similar radiopacities.

Micro-computed tomography, in addition to being a non-destructive method, allows the results to be compared or complemented with other diagnostic techniques in future work. As an example, in the present study, it was not possible to distinguish between cementum and dentin since they are two contiguous mineralized tissues with similar radiopacity. However, the fact that it allows acquisition in a wet state allows the future processing of teeth for histological analysis. Furthermore, the possibility of data storage allows the future reanalysis of morphological features of interest [29], using other specific algorithms, reducing the need to collect new samples for similar tests.

Despite its widespread use in clinical practice, intraoral radiography has limitations that may underestimate or provide insufficient information to accurately diagnose dental diseases [30]. Within the scope of this work, the acquisition of two phantoms with known radiodensity during the mCT analysis of the tooth, allows the relative MD to be determined with relative ease. The development of a software to calibrate and analyse dental radiographies obtained by using phantoms with known MD during the acquisition could pave the way to propose the dental X-ray as a non-invasive solution to calculate MD of teeth without needing extraction.

## 5. Conclusions

Micro-computed tomography was successfully employed to measure the mineral density of healthy feline teeth as well as to visualize their tridimensional morphology with definition and with a rotation amplitude of 360°. This knowledge could contribute to the development of new ancillary imagiological methods to diagnose and characterize dental disorders.

## Figures and Tables

**Table 1:** Mineral density of total tissues and hard tissues in the studied region of the tooth and its thirds.

Tooth	Region	Mean	Standard Deviation	Median	Maximum Value	Minimum Value	*p*
Total tissues	Total	1.374	0.040	1.365	1.436	1.323	0.0003
	Coronal	1.367	0.039	1.354	1.432	1.312	0.0275
	Middle	1.339	0.035	1.331	1.392	1.288	0.0038
	Apical	1.416	0.035	1.409	1.494	1.330	<0.0001
Hard tissues	Total	1.402	0.035	1.396	1.451	1.341	0.0009
	Coronal	1.393	0.041	1.408	1.450	1.315	0.0133
	Middle	1.372	0.031	1.372	1.415	1.320	0.0038
	Apical	1.441	0.038	1.436	1.505	1.389	<0.0001

## Data Availability

Not applicable.
